# Expansion of bone marrow‐derived human mesenchymal stem/stromal cells (hMSCs) using a two‐phase liquid/liquid system

**DOI:** 10.1002/jctb.5279

**Published:** 2017-04-24

**Authors:** Mariana P Hanga, Halina Murasiewicz, Andrzej W Pacek, Alvin W Nienow, Karen Coopman, Christopher J Hewitt

**Affiliations:** ^1^ Centre for Biological EngineeringLoughborough UniversityLoughboroughUK; ^2^ School of Chemical EngineeringUniversity of BirminghamBirminghamUK; ^3^ Aston Medical Research InstituteAston UniversityBirminghamUK; ^4^ West Pomeranian University of Technology SzczecinFaculty of Chemical Technology and EngineeringSzczecinPoland

**Keywords:** human bone marrow‐derived mesenchymal stem/stromal cells (hMSCs), two‐phase system, liquid/liquid interface, perfluorocarbons, expansion

## Abstract

**BACKGROUND:**

Human mesenchymal stem/stromal cells (hMSCs) are at the forefront of regenerative medicine applications due to their relatively easy isolation and availability in adults, potential to differentiate and to secrete a range of trophic factors that could determine specialised tissue regeneration. To date, hMSCs have been successfully cultured in vitro on substrates such as polystyrene dishes (TCPS) or microcarriers. However, hMSC sub‐cultivation and harvest typically employs proteolytic enzymes that act by cleaving important cell membrane proteins resulting in long‐term cell damage. In a process where the cells themselves are the product, a non‐enzymatic and non‐damaging harvesting approach is desirable.

**RESULTS:**

An alternative system for hMSC expansion and subsequent non‐enzymatic harvest was investigated here. A liquid/liquid two‐phase system was proposed, comprising a selected perfluorocarbon (FC40) and growth medium (DMEM). The cells exhibited similar cell morphologies compared with TCPS. Moreover, they retained their identity and differentiation potential post‐expansion and post‐harvest. Further, no significant difference was found when culturing hMSCs in the culture systems prepared with either fresh or recycled FC40 perfluorocarbon.

**CONCLUSIONS:**

These findings make the FC40/DMEM system an attractive alternative for traditional cell culture substrates due to their ease of cell recovery and recyclability, the latter impacting on overall process costs. © 2017 The Authors. *Journal of Chemical Technology & Biotechnology* published by John Wiley & Sons Ltd on behalf of Society of Chemical Industry.

## INTRODUCTION

Human mesenchymal stem/stromal cells (hMSCs) are at the forefront of regenerative medicine applications due to their relatively easy isolation and availability in adults, potential to differentiate into the three lineages (i.e. adipogenesis, chondrogenesis and osteogenesis) and the secretion of a range of trophic factors that could determine specialised tissue regeneration.^1^ Similarly to other adherent cell types, hMSCs can be expanded *in vitro* on solid cell culture substrates such as polystyrene dishes^2^, ^3^ or micrometer‐sized beads (i.e. microcarriers).^4^, ^5^, ^6^, ^7^ While cell attachment to substrates is advantageous for cell expansion, cell detachment from the same substrates may become a major challenge as cell viability and functionality post‐harvest has to remain intact. Cell detachment and subsequent recovery is at the moment probably one of the most important engineering factors that is hindering the faster development of cell‐based therapies. Over the years, numerous cell detachment strategies have been developed and these span a wide range of techniques adapted for different purposes and practical applications. Current cell harvesting methods make use of different cues, such as: mechanical (e.g. cell scraping,^8^ shear flow^9^), chemical (e.g. enzymes,^10^ chelators^11^), magnetic^12^ or even stimuli‐responsive (e.g. thermo‐responsive,^13^, ^14^ pH‐responsive,^15^ electro‐responsive,^16^, ^20^ photo‐responsive^17^).

The most common method for cell harvesting is enzyme digestion with particular reference to the proteolytic enzyme, trypsin or its derivatives. This method consists of the addition of an active concentration of enzyme to the cell culture system that works by cleaving the integrin–ligand bonds that mediate cell attachment to the given substrate.^18^, ^19^ This method provides an efficient way to break cell–surface and cell–cell interactions and is advantageous for applications requiring single cell suspensions rather than cell sheets. However, enzymatic treatment is an invasive approach and even though a promising technique has recently been developed,^20^ in the long term, it can potentially have damaging effects on the cells themselves by affecting critical cell membrane proteins.^18^, ^19^ A non‐enzymatic and non‐invasive harvesting approach could potentially offer advantages for the overall cell expansion process.

To overcome the deficiencies of the enzymatic method, alternative detachment methods have been developed. However, despite having some advantages over enzymatic treatments, these methods can be complicated, not necessarily scalable and potentially not of economic value. For example, electrochemically‐induced detachment can achieve cell detachment within minutes; however, it requires the elaborate fabrication of sensor features and often costly specialised coatings.^16^, ^21^ Another example of efficient cell detachment was from photo‐sensitive substrates resulting in >90% viable cells detached.^21^, ^22^ Despite their potential, this method can induce an irreversible modification of the substrate that impedes recycling of the material which can be expensive as it typically requires the synthesis of complicated substrates. Moreover, the use of UV as a stimulus for cell detachment could potentially have a mutagenic effect (dimerization of thymine molecules) on the cells.

Ideally, a simple, non‐complicated, non‐enzymatic, non‐invasive, scalable and cost‐effective approach to cell detachment should be implemented. As such, here we proposed and investigated a liquid/liquid two phase system comprising a selected perfluorocarbon and growth medium for the expansion and recovery of viable and fully functional hMSCs. As part of this study, we have recently published a paper on the process engineering aspects of using perfluorocarbon/DMEM systems for stem cell culture and this work builds on that earlier study.^23^ Perfluorocarbon liquids are fluorine‐substituted hydrocarbons with exceptional chemical and thermal stabilities, low surface tensions and specific gravities of about twice that of water.^23^ Perfluorocarbons have been intensively used in biomedical applications for almost two decades for biomedical imaging under the form of nano‐emulsions^24^, ^25^ or even biomedical therapeutics as an artificial, intravascular oxygen carrier.^26^, ^27^ Other biomedical applications include lung surfactant replacement^27^, ^28^ and ophthalmologic aids.^27^, ^29^ For cell and tissue culture systems, a series of benefits of using perfluorocarbons have been identified which include easy sterilisation, chemical and biological inertness, recoverability and recyclability, scavengers for gaseous cellular products and the ability to form two‐phase liquid/liquid systems.^30^, ^31^

When anchorage‐dependent cells are inoculated in the perfluorocarbon/medium two‐phase system, cells migrate to the interface where they attach, spread and grow. Several types of adherent cells have been successfully cultured to date, including fibroblasts,^32^, ^33^, ^34^ melanoma cells,^35^ epidermal carcinoma cells^32^ or myoblasts.^32^ The non‐enzymatic cell harvest approach when employing such a system consisted of simple pipetting,^34^ centrifugation^33^ or interface aspiration.^32^ The latter two are scalable to much larger systems. To the best of our knowledge, no studies on stem cell growth in a perfluorocarbon/medium two‐phase system have been published. Moreover, the potential of such a system to be used as a stem cell expansion and non‐enzymatic harvest system is dependent on whether stem cell quality is retained at the end of culture and post‐harvest.

## MATERIALS AND METHODS

### Cell culture on TCPS

Three human bone marrow‐derived human mesenchymal stem cells, identified here as M0, M2 and M4 (Lonza, Germany) were obtained from three different healthy donors of different age, sex and ethnicity (Supplementary Table 1), after the patient's informed consent. For all the experiments, hMSCs between passages 2 to 6 were used. The cells were cultured according to previously determined protocols.^2^, ^5^, ^7^ Briefly, the cells were seeded at 5000 cells cm^−2^ and grown on tissue culture plastic (TCPS) (NUNC, ThermoFisher, UK) in DMEM supplemented with 10% (v/v) foetal bovine serum and 2 mmol L^−1^ ultra‐glutamine (Lonza, UK). Growth medium was replaced every 3 days and the cells were passaged every 6 days. During passage, the hMSCs were first washed with Ca^2+^ and Mg^2+^ free phosphate buffered saline (PBS) (Lonza, UK). Cell detachment from the TCPS was achieved by incubating with 0.25% (w/w) trypsin/EDTA (Invitrogen, UK) for 7 min at 37°C and 5% CO_2_ in a humidified incubator. The enzyme was then inactivated by the addition of pre‐warmed growth medium and the cell suspension was centrifuged at 220 g for 5 min at room temperature. The obtained cell pellet was then resuspended in an appropriate volume of culture medium. Cells were counted and cell numbers were seeded as required for each experiment.

### Preparation of the liquid/liquid interface

Fluorinert FC40 (3M, Sigma Aldrich, UK) was used for the formation of the two‐phase systems to be used as cell culture substrates. The main physical properties of Fluorinert FC40 are listed in Table 1. Initially, the perfluorocarbon was sterilised by autoclaving at 121°C and 2 atm for 15 min. After use, the perfluorocarbon was recovered, recycled and re‐sterilised by vacuum filtration through a 0.2 µm filter unit (Millipore, UK). For all experiments, ultralow attachment 6 and 24‐well plates (Corning, UK) were employed to ensure cell attachment was only achieved on the tested substrates and not the plastic. For the preparation of the two‐phase systems, the perfluorocarbon FC40 was first added to the well plate, followed by the gentle addition of the growth medium so it formed a layer on top of the perfluorocarbon. A 1:2 ratio of perfluorocarbon to growth medium was used for all experiments. For example, in the 24‐well plates, 1 mL of perfluorocarbon was topped up with 2 mL of growth medium to ensure the formation of a flat interface.

**Table 1:** Main physical properties of the liquid phases involved in the formation of the two‐phase systems

Liquid	Chemical structure	Density (g mL^−1^)	Viscosity (at 25°C) (cSt)	Surface tension (dyn cm^−1^)
Fluorinert FC40	N(C_4_F_9_)_3_	1.85	2.2	16
DMEM	‐	1.02	1	72

### Cell culture on the liquid/liquid interface

The formation of the two‐phase system is based on mutual insolubility and density difference between the perfluorocarbon and the medium. Here the perfluorocarbon formed the bottom phase and the growth medium formed the top phase with cells growing at the interface. Before cell inoculation, the perfluorocarbon was conditioned in the growth medium at 37°C and 5% CO_2_ in a humidified incubator for at least 2 h. This conditioning step allows serum proteins to deposit on the liquid/liquid interface, thus facilitating cell adhesion to the flexible interface. hMSCs were again seeded at 5000 cells cm^−2^ of interfacial area and were kept in culture for up to 10 days depending on the experiment. Every 3 days in culture, a partial medium exchange was performed. For each individual experiment, a control experiment was conducted in parallel by culturing cells on TCPS without perfluorocarbon. For post‐expansion characterisation, the cells cultured on the interface of the FC40/DMEM system were harvested with trypsin to achieve a single cell suspension. Briefly, the spent medium was collected and replaced with pre‐warmed Ca^2+^ and Mg^2+^‐free PBS, followed by incubation in 1 mL of pre‐warmed 0.25% trypsin‐EDTA solution at 37°C and 5% CO_2_ for 5 min in a humidified incubator. The single cell suspension was collected and a cell pellet was obtained by centrifugation at 220 g for 5 min.

### Analytical techniques

Cell morphology was assessed by phase contrast on a Nikon Ti Eclipse microscope and by Live/Dead (Calcein AM/ Ethidium Homodimer) Viability/Cytotoxicity kit (Life Sciences, ThermoFisher, UK) visualized on a Nikon Ti Eclipse epi‐fluorescence microscope. The Live/Dead staining was performed following the manufacturer's instructions. Briefly, the cells were first washed with PBS and then incubated with the Live/Dead stain containing a final concentration of 2 µmol L^−1^ calcein‐AM and 4 µmol L^−1^ ethidium homodimer in the dark at 37°C and 5% CO_2_ for 40 min. After incubation, the Live/Dead working solution was removed and replaced with PBS. Cell counts and viability (via propidium iodide exclusion) were performed using the Nucleocounter NC‐3000 (Chemometec, UK). 1 mL spent medium samples were collected at different time points in culture to assess the metabolic activity of the cells in different conditions. The samples were stored at –20°C until analysis. When needed, medium samples were thawed, randomized and analysed for glucose, lactate and ammonia concentrations. Fresh growth medium samples were used as baseline control. Sample analysis was done on the Cedex Bio HT analyser (Roche, Germany). Based on cell counts and metabolic measurements, the following parameters were calculated:

1. Specific growth rate

(1) μ=lnCxtCx0Δt

where *μ* is the specific growth rate (h^−1^), *Cx*(*t*) and *Cx*(0) represent cell numbers at the end and start of the exponential growth, *t* represents time in culture (h).

- Doubling time

(2) $$td=\frac{\ln2}{\mu }$$

where *t_d_* is doubling time (h) and *μ* is the specific growth rate (h^−1^).

- Fold increase

(3) $$FI=\frac{Cx\left(f\right)}{Cx\left(0\right)}$$

where *Cx*(*f*) represents the maximum cell number and *Cx*(0) is the initial cell number.

- Specific metabolite production/consumption rate

(4) $$\text{qmet}=\frac{\mu }{Cx\left(0\right)}x\frac{\text{Cmet}\left(t\right)-\text{Cmet}\left(0\right)}{e^{\mu t}-1}$$

where *q_met_* represents the specific metabolite production/consumption rate (mol/cell/day), *Cmet(t)* and *Cmet*(0) are the metabolite concentrations at the end and the start of the exponential growth, respectively, *Cx*(0) represents the cell number at the start of the exponential growth phase and *t* is time in culture (h).

### Proliferation assessment

For this experiment, only M0 and M2 cell lines were used for proliferation assessment. During the last 24 h culture, the cells were pulsed with EdU (5‐ethynyl‐2'‐deoxyuridine). EdU is a nucleoside analogue of thymidine and is incorporated into DNA during active DNA synthesis, thus allowing for the detection of cells entering the S‐phase of their cycle. After 72 h, the culture was stopped and the cells were processed following the manufacturer's protocol for the Click‐iT EdU AlexaFluor 488 kit (Life Technologies, ThermoFisher, UK). Imaging was performed on a Nikon Ti Eclipse fluorescence microscope. EdU quantification was done using the ImageJ software (NIH) by counting the number of DAPI stained nuclei (blue) and nuclei positive for EdU (green). The results were averaged from three independent experiments and a minimum of 300 nuclei were counted for each condition.

### Immuno‐phenotype analysis

Immuno‐phenotype analysis was performed before and after hMSC expansion on the FC40/DMEM interface. hMSC immune‐phenotype was determined by multi‐parameter flow cytometry by using a previously developed protocol.^3^ Briefly, cells were harvested from the substrate as described previously, counted and the cell suspension concentrated to achieve 1 × 10^6^cells mL^−1^. 200 μL of cell suspension was then loaded onto a V‐bottom 96 well plate and centrifuged at 220 g for 5 min. The supernatant was then aspirated, the cell pellet resuspended in flow cytometry staining buffer (R&D Systems, UK) and centrifuged again. The cells were stained with mouse anti‐human monoclonal antibodies (BD Biosciences, UK) for 30 min in the dark at room temperature. The fluorescently‐labelled antibodies were selected based on a panel recommended by the International Society for Cell Therapy (ISCT)^22^ and these were: CD73 (PE‐Cy7), CD90 (APC), CD105 (PE), CD34 (PE‐Cy5) and HLA‐DR (FITC). After incubation, the samples were washed twice with staining buffer as described before. The stained samples were then analysed on the Guava EasyCyte 8HT flow cytometer (Merck Millipore, UK) equipped with 488 nm and 640 nm excitation lasers. A minimum of 10 000 gated events were recorded for each sample. Post‐acquisition analysis and compensation were performed using the FlowJo software (Treestar Inc, USA).

### Immunofluorescence staining

For immunofluorescence experiments, cells were seeded at 10 000 cells cm^−2^ of interfacial/surface area. At the desired time points in culture, cells were fixed with 4% (v/v) para‐formaldehyde at room temperature for 30 min, followed by washing twice with staining buffer (Biolegend, UK). Non‐specific binding and staining was blocked with blocking buffer containing 10% v/v normal goat serum (ThermoFisher, UK) at room temperature for 45 min. The blocking buffer was then removed and the rabbit anti‐paxillin primary antibody (Y113) (Abcam, UK) was added at a 1:100 dilution and incubated at 2–8°C overnight. The primary antibody solution was then removed, samples washed twice with staining buffer. The goat polyclonal secondary antibody to rabbit IgG (AlexaFluor 488 conjugated) (Abcam, UK) was then added at a 1:200 dilution and incubated at room temperature for 2 h. Following two washes, the actin filaments were stained with AlexaFluor‐546 phalloidin (Life Technologies, ThermoFisher, UK) following the manufacturer's protocol. Cells were incubated in the Phalloidin working solution at room temperature in the dark for 20 min, followed by two washes with PBS. Nuclei were stained with DAPI (Life Technologies, ThermoFisher, UK) (300 nmol L^−1^ working solution) at room temperature in the dark for 5 min. Samples were visualised on a Nikon Ti Eclipse fluorescence microscope.

### Differentiation potential assessment

All chemicals were obtained from Sigma Aldrich (UK) unless otherwise stated. Differentiation studies were performed before and after cell expansion on the FC40/DMEM interface by following well established protocols.^3^, ^7^ hMSC differentiation into the three lineages was induced by using the complete differentiation kits StemPro (ThermoFisher, UK). Briefly, for adipogenic and osteogenic differentiation, cells were seeded in growth medium at 5000 cells cm^−2^ in 12‐well plates, allowed to attach and proliferate for 24–48 h until 70–80% confluency was achieved and then the growth medium was replaced with differentiation medium. For chondrogenic differentiation, a cell pellet was generated by centrifugation at 220 g for 5 min, followed by re‐suspension to achieve a 1 × 10^7^ cells mL^−1^ concentration. 5 μL droplets of the concentrated cell suspension were then plated in empty 12‐well plates and incubated at 37°C and 5% CO_2_ in a humidified incubator for 1–2 h to allow cell attachment, after which 2 mL of chondrogenesis differentiation medium was gently added to the wells to avoid disturbing the macro‐masses. Differentiation medium was changed every 3–4 days and cells were cultured for 21 days. After 21 days, cells were washed and fixed for 30 min with 4% (v/v) para‐formaldehyde at room temperature. Adipocytes were stained with Oil Red O. Briefly, initially a stock solution of 0.3% (w/v) Oil Red O in 99% isopropanol was prepared. For adipocyte staining, 3 parts of the Oil Red O stock was mixed with 2 parts distilled water and incubated at room temperature for 10 min, followed by two washes with distilled water. Chondrocytes were stained with 1% (w/v) Alcian Blue in 0.1 N HCl solution for 60 min, washed twice with 0.1 N HCl and once with distilled water. To evaluate osteogenic differentiation, cells were first stained for Alkaline Phosphatase (ALP) at room temperature in the dark for 45 min using a solution containing Fast Violet B salts with 4% (v/v) naphtol AS‐MX phosphate alkaline solution. Calcium mineralization was stained with 2.5% (w/v) silver nitrate at room temperature in the presence of light for 30 min. After staining, cells were washed three times with distilled water and then visualised under a Nikon Ti Eclipse phase contrast microscope.

### Colony forming unit fibroblast (CFU‐F) assay

Colony forming unit efficiency of hMSC before and after expansion on the FC40/DMEM interface was determined employing a protocol previously developed.^5^ Briefly, harvested hMSCs were seeded at 10 cells cm^−2^ in T‐25 flasks and kept in culture in normal growth conditions for up to 14 days. Complete medium changes were done every 3–4 days. After 14 days, the colonies were fixed for 30 min at room temperature with 4% (v/v) para‐formaldehyde and then stained with 1% (w/v) Crystal Violet solution at room temperature for 60 min. Colonies of 25 cells and higher were then counted and CFU‐F efficiency (%) was calculated using Equation (5).

(5) $$CFU-F\;\text{Efficiency}\;\left(\%\right)=\left(\frac{250}{\text{Number of Colonies}}\right)\cdot 100$$

Statistical analysis was performed by SPSS (IBM, USA) using either paired‐sample t‐tests or two‐way ANOVA. Results were deemed to be significant if *P*‐value <0.05.

## RESULTS AND DISCUSSION

### hMSC morphology when cultured on the FC40/DMEM interface

**Figure 1:**
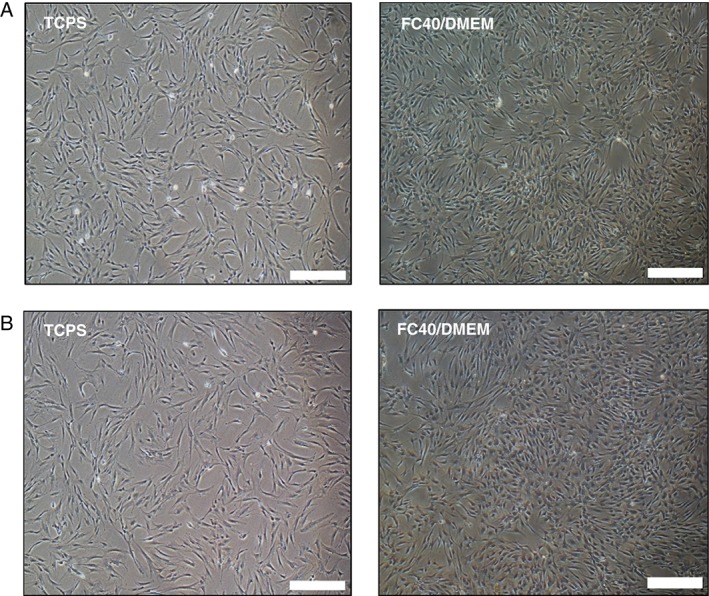
(A) M0 and (B) M2 hMSCs seeded at 5000 cells cm^−2^ on TCPS and the FC40/DMEM interface, after 72 h in culture. Scale bar represents 500 µm.

**Figure 2:**
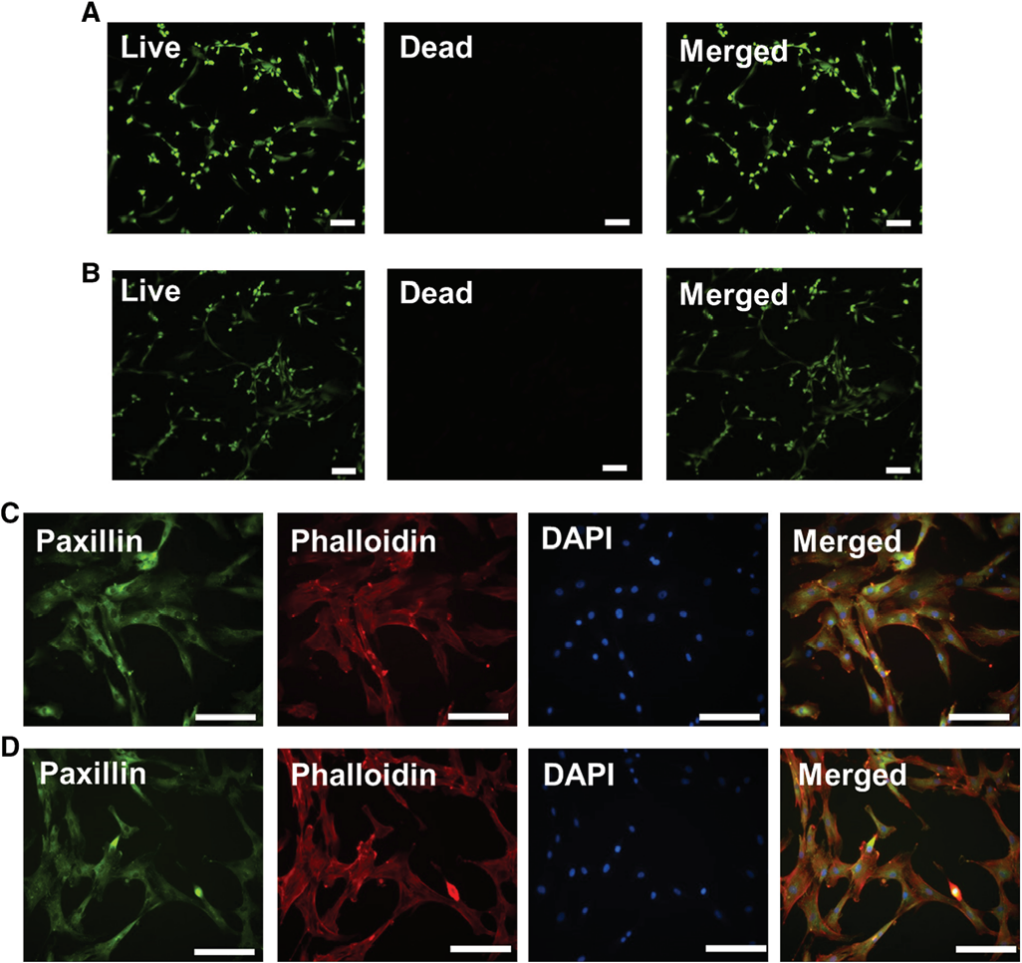
M2 cells seeded at 10 000 cells cm^−2^. Live/Dead staining (green – live and healthy cells, red – dead or damaged cells) of cells at day 2 in culture on (A) TCPS, (B) FC40/DMEM interface. Immunocytochemistry staining of cells cultured on (C) TCPS and (D) FC40/DMEM interface. Nuclei staining with DAPI (blue), actin filaments with Phalloidin (red) and focal adhesions with paxillin (Green). Scale bar represents 100 µm.

When inoculated in the FC40/DMEM system, hMSCs localized at the interfacial area between the two immiscible liquids, at the hydrophilic side of the interface. No cells were observed at the hydrophobic side. The same behaviour was previously reported in studies involving other cell types such as fibroblasts.^32^, ^34^ After conditioning the interfacial area in the serum‐containing DMEM for at least 2 h before cell inoculation, hMSCs attached and exhibited the typical spindle‐like morphology characteristic to MSCs, similarly to traditional plastic surfaces (Fig. 1). Moreover, the cells remained viable (viability >90%) when cultured on the FC40/DMEM interface with only a few dead cells, as seen by Live/Dead staining and fluorescence imaging. Live and healthy cells are green with an elongated fibroblast‐like morphology, while dead or damaged cells are rounded and are stained red (Fig. 2(A) and (B)). In addition, immunostaining of the actin filaments and the focal adhesion protein, paxillin was performed to assess if any changes in hMSC morphology or cell–matrix interactions occur when cultured on the FC40/DMEM interface compared with TCPS. However, in both cases, the hMSCs appeared well spread with accumulated punctate paxillin around the nucleus and distinct actin stress fibres around the peripheral edges, as shown in Fig. 2(C) and (D) for M2 cell line and in Supplementary Fig. 1 for M0 cell line.

### hMSC growth on the flexible liquid/liquid interface

**Figure 3:**
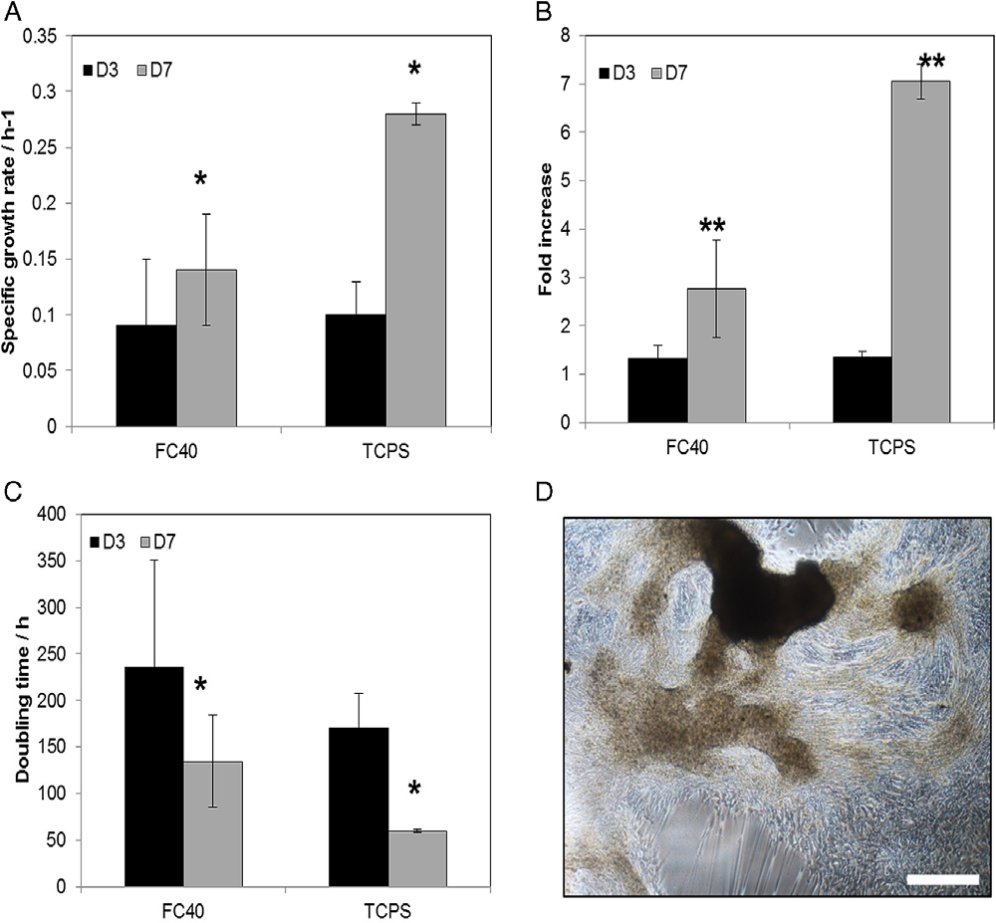
M2 hMSC donor line growth kinetics over 7 days in culture on the FC40/DMEM interface compared to TCPS. Shown as (A) Specific growth rate, (B) fold increase, (C) doubling time. Error bars represent the standard deviation of two independent experiments. Statistical analysis by paired‐sample t‐test * P < 0.05; ** P < 0.005; (D) Unwanted cell sheet detachment and folding at day 6 in culture resulting in hMSC aggregates. Scale bar represents 500 µm.

**Figure 4:**
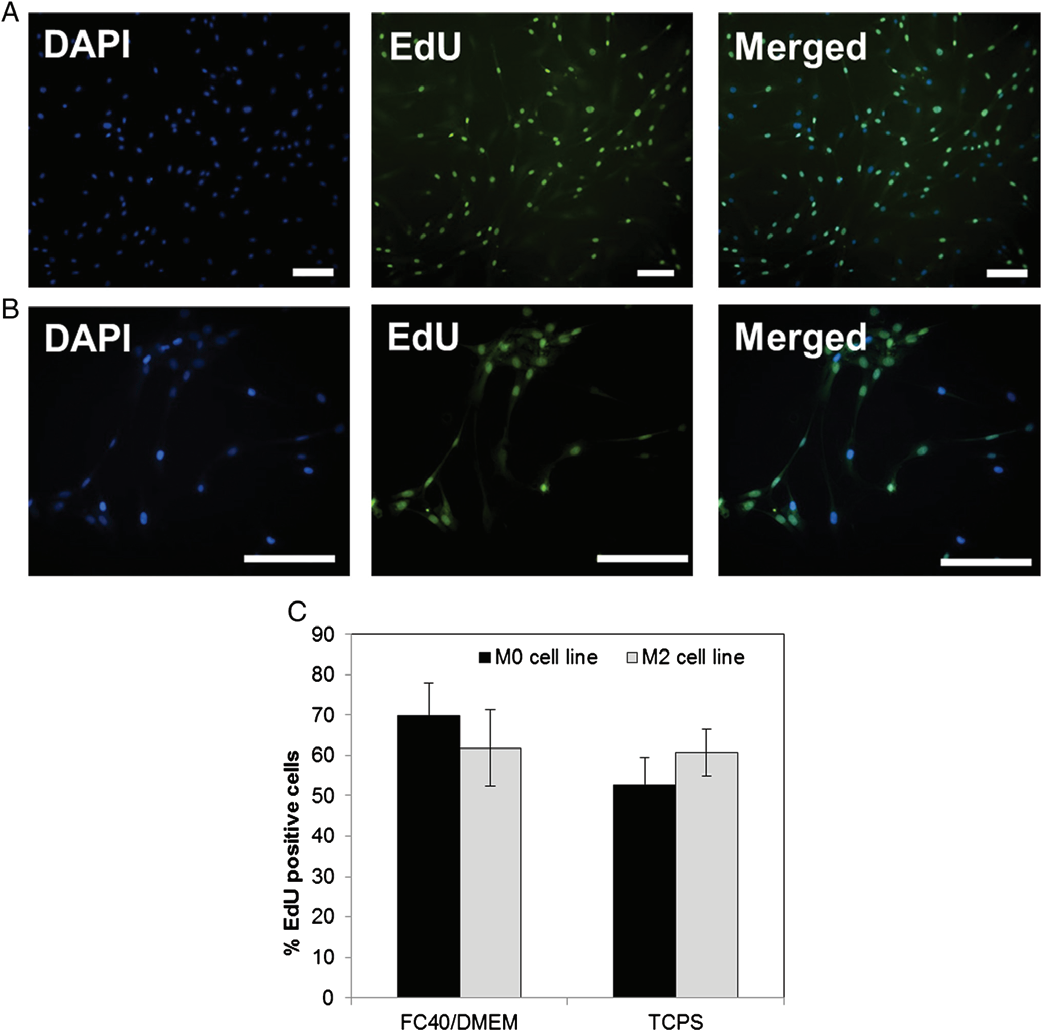
Representative fluorescent images of M2 cells cultured for 3 days on (A) TCPS and (B) FC40/DMEM interface after being pulsed with EdU for the last 24 h culture. Scale bars represent 100 µm. (C) Percentage of cells entering S‐phase as quantified by EdU staining. Data expressed as Mean ± SD; N > 300 nuclei counted.

M2 cells were expanded on the flexible FC40/DMEM interface and kept in culture for up to 7 days. In parallel, M2 cells at the same passage number were cultured on traditional solid substrates provided by TCPS and kept in culture for the same time period. At day 3 in culture, no significant difference was observed in cell growth on the two tested substrates. As such, a 1.33 ± 0.26‐fold increase was achieved on the FC40/DMEM interface compared with 1.36 ± 0.11‐fold increase when cultured on TCPS (Fig. 3(B)). Moreover, it was observed that the growth kinetics were similar with a specific growth rate of 0.091 ± 0.063 day^−1^ in the FC40/DMEM system compared with 0.102 ± 0.025 day^−1^ on TCPS (Fig. 3(A)). In addition, the percentage of EdU‐positive cells indicating the number of proliferative hMSCs at day 3 in culture was slightly higher when cultured in the FC40/DMEM system compared with TCPS, as shown in Fig. 4. However, by day 7 in culture, the final fold expansion increase was 2.77 ± 1.01 on the FC40/DMEM interface, but 2.5 times higher on the traditional TCPS (p < 0.005) (Fig. 3(B)). The same behaviour was seen in the growth kinetics of M2 hMSCs on the two substrates: a specific growth rate of only 0.14 ± 0.05 day^−1^ was achieved in FC40/DMEM, 2 times lower than the specific growth rate on TCPS for the same culture period (*P* < 0.05). In addition, a significant increase (*P* < 0.05) in doubling times was found when cultured in the two‐phase system, 134.4 ± 49.6 h compared with culture on TCPS, 59.7 ± 1.6 h (Fig. 3(C)).

Lower cell yields when cultured on the perfluorocarbon/DMEM interfaces have previously been found.^32^, ^34^ However, different cell types and even perfluorocarbons were used and to the best of our knowledge, no previous studies have been done using hMSCs or stem cells to investigate cell attachment and growth in a perfluorocarbon/DMEM two‐phase system. Moreover, hMSCs as defined by the ISCT criteria^36^ are adherent cells that preferentially adhere to surfaces such as TCPS. In addition, it has been found that when cultured on soft polyacrylamide gels that mimic the elasticity of bone marrow and fat tissues, bone marrow‐derived hMSCs became quiescent; however, they resumed proliferation once replated on TCPS.^36^ On the contrary, despite the lower cell growth rates calculated when grown in the FC40/DMEM system, we found that bone marrow‐derived hMSCs attached and proliferated on the FC40/DMEM interface. The lower cell yields measured at the end of the culture could be a result of unwanted cell sheet detachment and folding, resulting in the formation of hMSC aggregated areas as seen in Fig. 3(D). As the interface becomes populated with cells, the protein layer deposited on the interface together with the extracellular matrix produced by the cells becomes stretched and deformed resulting in unwanted cell sheet detachment and folding and the formation of hMSC aggregates. Depending on the size of aggregates formed, it is believed that the proliferation capacity of the cells becomes reduced,^38^, ^39^, ^40^ thus possibly explaining the lower cell yields obtained at the end of culture in the FC40/DMEM system.

**Figure 5:**
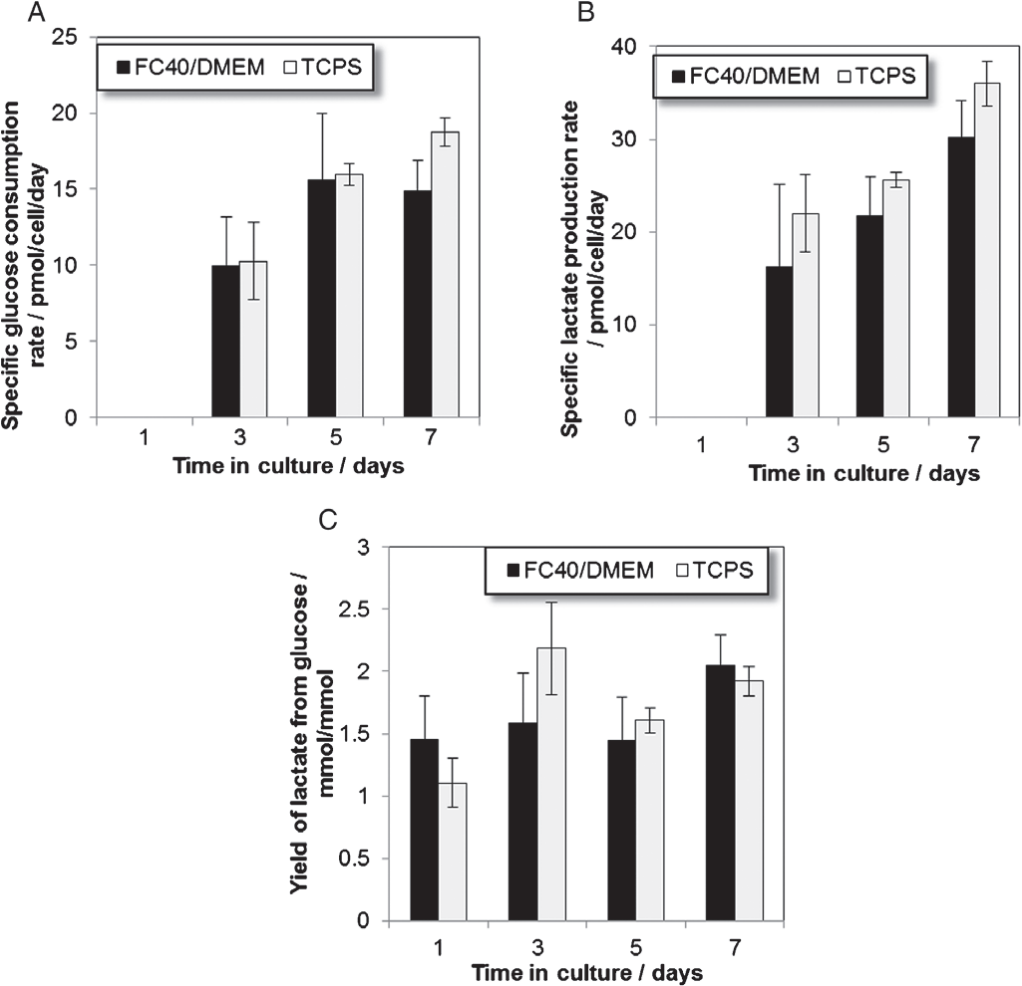
Nutrient and metabolite flux of hMSC M2 donor cell line expanded over 7 days on the FC40/DMEM interface and traditional TCPS. (A) Specific glucose consumption rate; (B) Specific lactate production rate and (C) Yield of lactate from glucose. Error bars represent the standard deviation of two independent experiments.

To investigate the cell growth rates further, nutrient usage and metabolite production during culture on the two different substrates was measured by spent medium concentration analysis for glucose, lactate and ammonia. Statistical significance was assessed by two‐way ANOVA. When cultured on both substrates, glucose consumption increased significantly with time in culture (P < 0.0001), however, no significant difference (P > 0.05) was found in glucose consumption when M2 cells were cultured on the two substrates for 7 days (Fig. 5(A)). On the other hand, lactate production also increased significantly with time in culture in both scenarios (P < 0.0001), however, lactate production was significantly higher (P < 0.05) when the M2 cells were cultured on TCPS compared with the FC40/DMEM interface (Fig. 5(B)).

Moreover, the estimated yield of lactate from glucose over the entire culture period was found to be 2.04 ± 0.25 mol_lactate_/mol_glucose_ and 1.92 ± 0.12 mol_lactate_/mol_glucose_, respectively, with no significant difference when cultured on the FC40/DMEM interface or TCPS (*P* > 0.05), respectively (Fig. 5(C)).

The yield of lactate to glucose represents a measure of how efficiently cells metabolize glucose into energy. As such, there are two possible metabolic pathways for glucose consumption and these are either oxidative phosphorylation yielding 30 to 38 moles of ATP per mole of glucose or anaerobic glycolysis which yields 2 moles of lactate from one mole of glucose.^41^, ^42^ The calculated Y_lac/glc_ values of around 2 mol/mol at the end of culture suggested that the preferred metabolic pathway was the anaerobic glycolysis when cultured on either of the tested substrates. However, when looking at the lactate from glucose yield produced at different time points during culture, it was found that actually a combination of oxidative phosphorylation and anaerobic glycolysis was involved in glucose metabolism. As such, regardless of the tested substrate, at the beginning of the culture, at lower cell growth rates, the cells consumed glucose in a more efficient manner for energy production, as suggested by the Y_lac/glc_ values closer to 1 mol/mol (Fig. 5(C)). Despite the lower cell numbers at the beginning of the culture, the high energy produced by the cells is not used for cell growth alone, but also for basic cell maintenance functions.^42^, ^43^ The slight increase in the Y_lac/glc_ values at day 3 in culture suggesting lower energy production rates could be attributed to the accumulation of metabolites and consumption of nutrients, while the decrease in Y_lac/glc_ and the more efficient energy production from glucose after 3 days in culture could be possibly attributed to the partial medium exchange done at day 3.

Another parameter that was measured during the culture on the two substrates was ammonia production. Ammonia is mainly released by metabolic deamination of glutamine to glutamate and conversion of glutamate to α‐keto‐glutarate.^44^ Ammonia can also be released by spontaneous decomposition of L‐glutamine. However, this cannot be the case here as we use a stable form of glutamine in our cultures represented by ultra‐glutamine. Several previous studies have suggested that ammonia build‐up in the medium could potentially have a growth inhibitory effect at relatively low concentrations. For example, a concentration of approximately 3 mmol L^−1^ of ammonia was found to be growth inhibitory for goat‐sourced MSCs.^42^ As such, another possible explanation for the lower cell growth rates when cultured on the flexible liquid/liquid interface might be the accumulation of ammonia. However, this was not the case here as the highest ammonia concentration recorded at the end of culture was 0.27 mmol L^−1^.

### Effect of perfluorocarbon recyclability on hMSC proliferation on the FC40/DMEM interface

One of the major advantages of using perfluorocarbons as cell culture systems could be the ability to recover and recycle them.^30^ This benefit could have great implications in the large scale by reducing manufacturing costs. However, the first thing to test was the effect of recovering and recycling the perfluorocarbon, as well as repeated sterilisation on hMSC attachment and growth. Filter‐sterilised, unused perfluorocarbon was referred to as FC40_fresh_, while previously used, recovered and recycled filter‐sterilised perfluorocarbon was referred to as FC40_recycled._ As such, hMSCs were seeded as before at 5000 cells cm^−2^ on the interfaces created in the FC40_fresh_/DMEM and FC40_recycled_/DMEM systems and kept in culture for up to 6 days. To account for donor variability,^45^, ^46^ three hMSC lines originating from three different donors (nomenclature presented in Supplementary Table 1) were employed for this part of the study.

**Figure 6:**
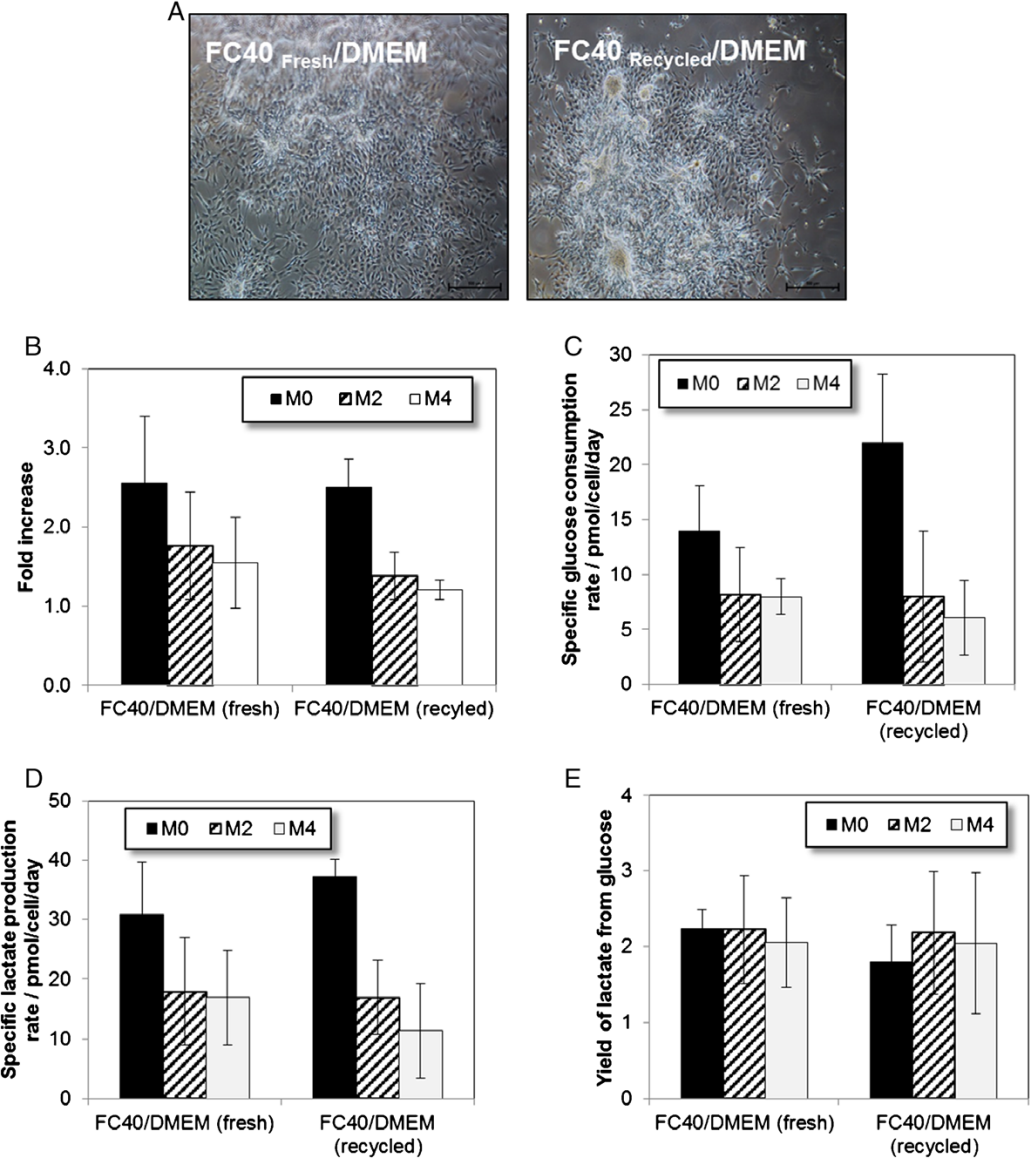
Data for culturing on the FC40fresh/DMEM or FC40recycled/DMEM interfaces after 6 days (A) Representative phase contrast images of M2 cell morphology. Scale bar represents 500 µm. Quantitative data for M0, M2 and M4 donor cell lines. (B) Fold increase; (C) specific glucose consumption rate, (D) specific lactate production rate and (E) yield of lactate from glucose. Error bars represent the standard deviation of two independent experiments.

Regardless of the cell line used, hMSCs maintained their cell morphology when cultured on either FC40_fresh_/DMEM or FC40_recycled_/DMEM interfaces_._ Figure 6(A) shows representative images for one of the selected hMSC lines (i.e. M2 cell line). Images showing no evident difference in M0 and M4 cells morphology when cultured on either fresh or recycled FC40/DMEM interface are presented in Supplementary Fig. 2. Moreover, independently of the cell line used, there was no significant difference in hMSC growth on either fresh or recycled FC40/DMEM interface (Fig. 6(B)). No significant difference was found in the specific glucose consumption rates (Fig. 6(C)) or the specific lactate production rates (Fig. 6(D)) of any of the three hMSC lines tested. In addition, at the end of the culture, no significant difference was found in the calculated Y_lac/glc_ values and moreover, the values of around 2 mol_lactate_ /mol_glucose_ suggested that the preferred metabolic pathway of all hMSC lines tested was the anaerobic glycolysis when cultured on either of the tested substrates (Fig. 6(E)). These findings of no significant differences in cell growth on the liquid/liquid interfaces made with either fresh or recovered and recycled perfluorocarbon could have major implications for the production costs at large scales as a result of the recoverability and recyclability of the perfluorocarbon. Also, donor variability did not seem to have an impact on cell attachment and growth on either the fresh or recycled FC40/DMEM interfaces and the growth profiles of the selected hMSC lines on the FC40/DMEM interfaces were similar to traditional TCPS when cultured as monolayers, with M0 being the fastest growing and M4 being the slowest growing as found previously by us.^2^

### Post‐expansion characterisation

**Figure 7:**
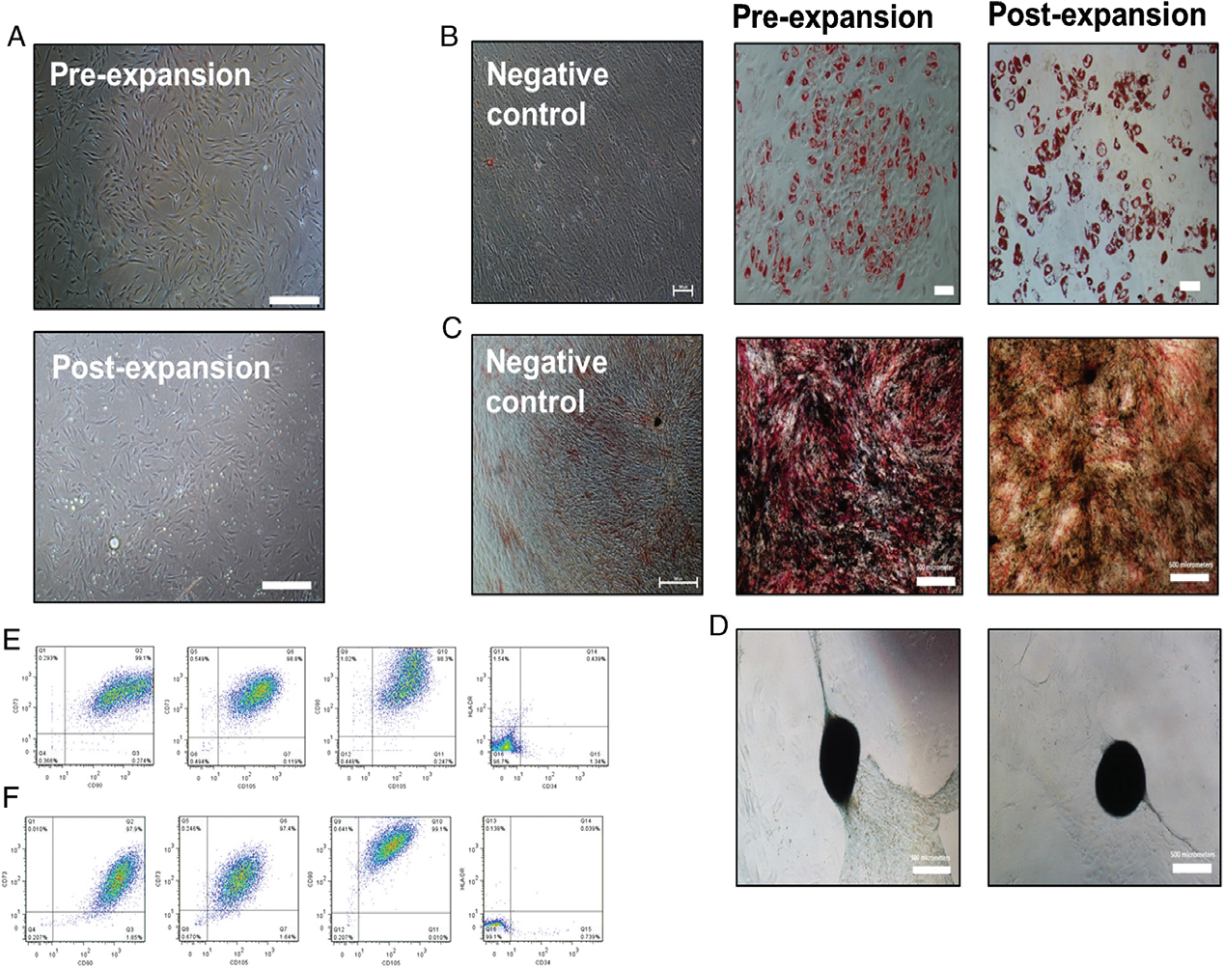
Pre‐ and post‐expansion of hMSC M2 cell line characterisation. (A) Cell morphology. Scale bars represent 500 µm. Tri‐lineage differentiation: (B) adipogenesis (scale bar 100 µm), (C) osteogenesis (scale bar 500 µm) and (D) chondrogenesis (scale bar 500 µm). Multi‐parameter flow cytometry analysis showing dual gating of CD73, CD90, CD105, CD34 and HLA‐DR for M2 hMSCs (E) pre‐expansion and (F) post‐expansion.

hMSC characterisation was performed post‐expansion at the FC40/DMEM interface to ensure that the expansion on such a substrate did not have a detrimental effect on the hMSC identity and quality. As such, the International Society of Cellular Therapy (ISCT) criteria were applied.^36^ Post‐expansion characterisation was performed for all three hMSC lines cultured on the FC40/DMEM interface. A representative example of conformance to the ISCT criteria is shown for the M2 cell line pre‐ and post‐expansion in Fig. 7. hMSCs adhered to TCPS post‐harvest and post‐expansion for 6 days on the FC40/DMEM interface and exhibited similar cell morphologies to pre‐expansion (Fig. 7(A)). Moreover, the M2 hMSCs differentiated down the adipogenic (Fig. 7(B)), osteogenic (Fig. 7(C)) and chondrogenic (Fig. 7(D)) lineages, both pre‐ and post‐expansion on the FC40/DMEM interface. The stiffness of the surface on which MSCs are cultured is known to affect their lineage potential,^47^ however, as assessed by histology, the brief culture period on the ‘soft’ FC40/DMEM interface has not affected the ability of the MSCs to differentiate into any of the trilineages once replated back onto TCPS. Figure 7(E) and (F) show the pre‐expansion and post‐expansion immunophenotype of hMSCs, respectively. In both cases, the co‐expression of positive cell surface markers (e.g. CD73, CD90 and CD105) were greater than 97%, while the expression of the negative markers (e.g. CD34, HLA‐DR) was found to be below 2%, in accordance with the ISCT criteria for identifying the MSC immunophenotype.^36^ Similarly, the other tested hMSC lines, M0 and M4, retained the same cell morphology as seen on TCPS after expansion for 6 days on the flexible FC40/DMEM interface (Supplementary Fig. 2). Moreover, post‐expansion, the M0 and M4 cells also retained their differentiation potential down the adipogenic and osteogenic lineages, demonstrated the co‐expression of the positive cell surface markers, CD73, CD90 and CD105 and lacked the expression of the negative markers, CD34 and HLA‐DR (Supplementary Figs 3 and 4).

**Figure 8:**
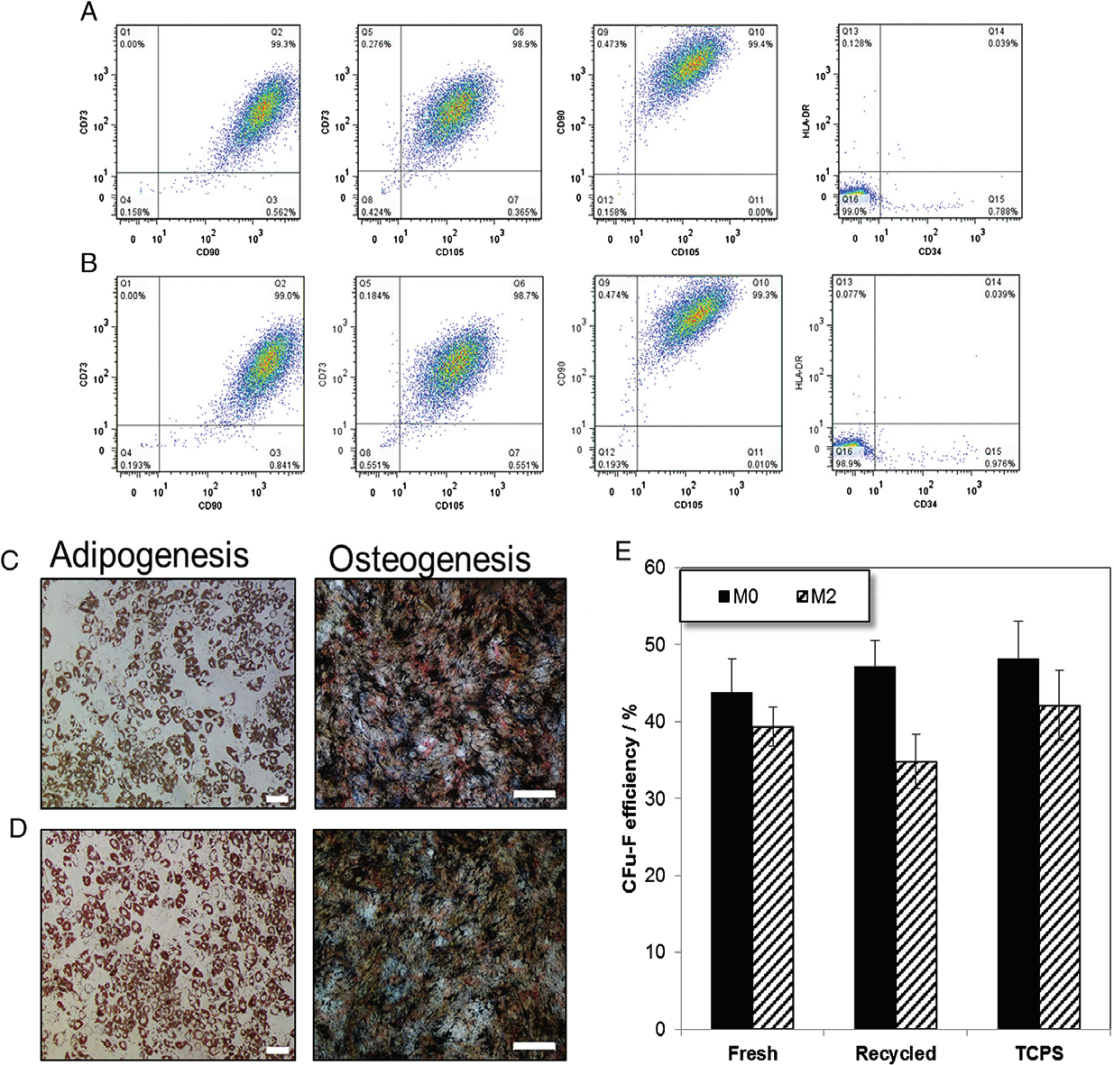
Post‐expansion characterisation after 6 days of M2 hMSCs. Multiparameter flow cytometry (A) FC40_fresh_/DMEM and (B) FC40_recycled_/DMEM. Differentiation potential: adipogenesis (Scale bar 100 µm) and osteogenesis (Scale bar 500 µm) (C) FC40_fresh_/DMEM and (D) FC40_recycled_/DMEM system. (E) Colony forming unit‐fibroblast (CFU‐F) efficiency for M0 and M2 lines. Data expressed as Mean ± SD (N = 6).

No significant difference was found in hMSC growth in either the FC40_fresh_/DMEM or FC40_recycled_/DMEM systems with possible cost implications at larger scales. However, the successful development of hMSC manufacturing system is deemed successful if cell quality and identity remain intact.^5^, ^48^ As such, it was imperative to determine if hMSC identity and quality were affected by the perfluorocarbon recycling step. Figure 8(A) and (B) show the multiparameter flow cytometry analysis of harvested M2 cells after 6 days culture in the two‐phase systems when using either fresh or recycled perfluorocarbon, demonstrating the co‐expression and lack of expression of the cell surface markers panel recommended by the ISCT. Moreover, the hMSCs retained their differentiation potential post‐expansion from both phase systems, FC40_fresh_/DMEM (Fig. 8(C) and FC40_recycled_/DMEM (Fig. 8(D)), respectively.

Colony forming unit potential has been previously highlighted as an important hMSC quality parameter^49^ and it has been shown to deteriorate in certain conditions.^50^, ^51^ Figure 8(E) shows the colony forming unit‐fibroblast (CFU‐F) efficiency of the M0 and M2 cell lines when cultured on either TCPS or the FC40_fresh_/DMEM or FC40_recycled_/DMEM interfaces. No significant difference was found in the CFU‐F efficiency of M0 line when cultured in either FC40_fresh_/DMEM or FC40_recycled_/DMEM systems. However, a significant difference (*P* < 0.05) was found in the CFU‐F efficiency of M2 line when expanded on the two substrates tested (Fig. 8(E)). This difference could possibly be attributed to donor variability and be related to cell growth kinetics.

## CONCLUSIONS

The liquid/liquid system tested here is a promising candidate for the development of novel stem cell expansion systems. The presence of cells on the hydrophilic side of the flexible interface leads to the development of a scalable enzyme‐free harvesting method for cell sheet engineering. Moreover, cell harvest from the proposed system could be performed without the use of enzymes by simply aspirating, centrifuging or filtering the interface resulting in the recovering of cells with intact membrane proteins. The proposed system is simple, ready‐to‐use and does not require any modification or coating in order to support MSC attachment and proliferation. Moreover, MSCs cultured on the flexible interface for up to 7 days retained their potency and identity post‐expansion. In addition, the FC40/DMEM system can be employed at larger scales by dispersing the perfluorocarbon into the growth medium in the form of droplets that act as ‘liquid microcarriers’ for cell expansion.^23^ In addition, this system has the potential to be cost‐effective when employed at larger scales as a result of the perfluorocarbon recoverability and recyclability. There was no significant difference in cell attachment, growth or even cell quality when cultured on the interface prepared with recycled perfluorocarbon compared with fresh, not previously used perfluorocarbon. Previous reports suggested that MSCs become quiescent when cultured on a soft gel‐like substrate with resuming proliferation when transferred to TCPS. As such, MSC proliferation on the FC40/DMEM interface was unexpected. As a result, the proposed FC40/DMEM system could also find applicability for cell–substrate interactions or cell migration studies.

## CONFLICT OF INTEREST

The authors declare that there is no financial or commercial conflict of interest.

## Supporting information


**Supplementary Figure 1.** Immunocytochemistry staining for M0 cells at Day 2 in culture (A) TCPS and (B) FC40/DMEM interface. Green – anti‐paxillin staining; Red – Phalloidin staining; Blue – DAPI. Scale bar represents 100 µm.
**Supplementary Figure 2.** Phase contrast images of (A) M2 and (B) M4 cell morphology at day 2 in culture on FC40fresh/DMEM or FC40recycled/DMEM interfaces. Scale bar represents 500 µm.
**Supplementary Figure 3.** Cell surface markers expression of M0 cell line cultured on (A) fresh and (B) recycled FC40/DMEM interfaces, assessed by multi‐parameter flow cytometry.
**Supplementary Figure 4.** Cell surface markers expression of M4 cell line cultured on (A) fresh and (B) recycled FC40/DMEM interfaces, assessed by multi‐parameter flow cytometry.
**Supplementary Table 1.** Summary of the bone marrow derived human mesenchymal stem cell lines used in this study – nomenclature and donor information
