# Supplementary material for: Expansion of bone marrow‐derived human mesenchymal stem/stromal cells (hMSCs) using a two‐phase liquid/liquid system
Source: J Chem Technol Biotechnol. 2017 Apr 24;92(7):1577–89. doi: 10.1002/jctb.5279 (PMC5485050; doi:10.1002/jctb.5279)
Supplement: Supplementary file 1 — Supplementary Figure 1. Immunocytochemistry staining for M0 cells at Day 2 in culture (A) TCPS and (B) FC40/DMEM interface. Green – anti‐paxillin staining; Red – Phalloidin staining; Blue – DAPI. Scale bar represents 100 µm. Supplementary Figure 2. Phase contrast images of (A) M2 and (B) M4 cell morphology at day 2 in culture on FC40fresh/DMEM or FC40recycled/DMEM interfaces. Scale bar represents 500 µm. Supplementary Figure 3. Cell surface markers expression of M0 cell line cultured on (A) fresh and (B) recycled FC40/DMEM interfaces, assessed by multi‐parameter flow cytometry. Supplementary Figure 4. Cell surface markers expression of M4 cell line cultured on (A) fresh and (B) recycled FC40/DMEM interfaces, assessed by multi‐parameter flow cytometry. Supplementary Table 1. Summary of the bone marrow derived human mesenchymal stem cell lines used in this study – nomenclature and donor information [file JCTB-92-1577-s001.docx]

*Supplementary Table 1.* Summary of the bone marrow derived human mesenchymal stem cell lines used in this study – nomenclature and donor information

| **BM-hMSC line nomenclature** | **Donor Age** | **Donor Sex** | **Donor Ethnicity** |
| --- | --- | --- | --- |
| M0 | 20 | Male | Black |
| M2 | 19 | Female | Black |
| M4 | 25 | Female | Hispanic |


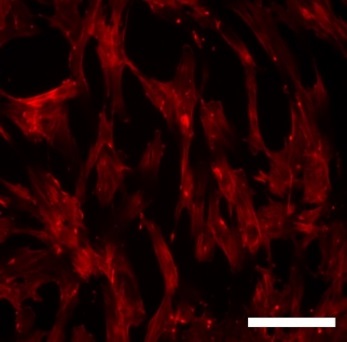

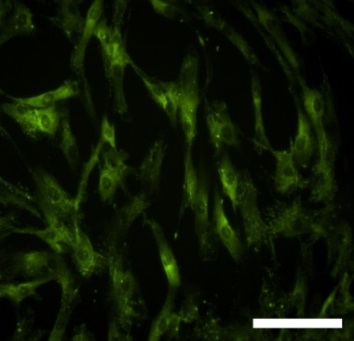

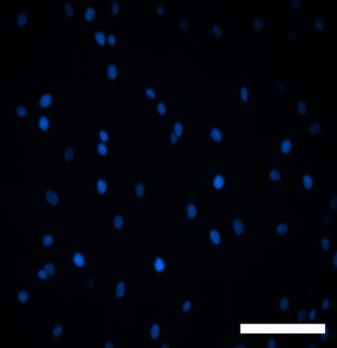

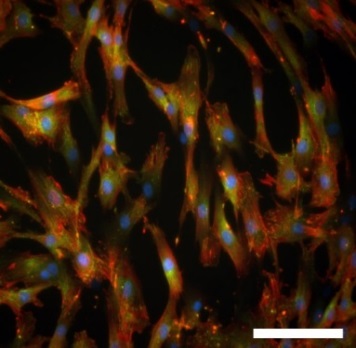


**Paxillin**

**Phalloidin**

**Merged**

**A)**

**B)**

**DAPI**


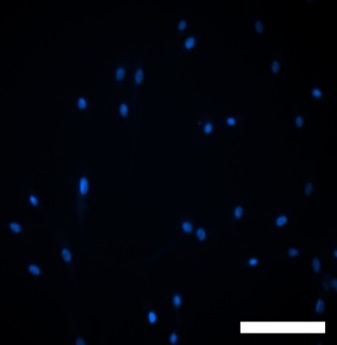


**DAPI**


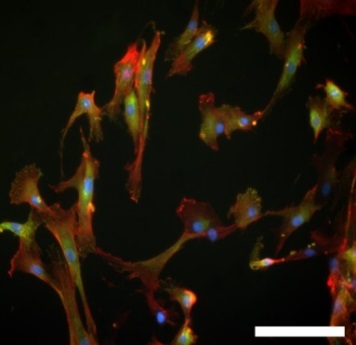


**Merged**


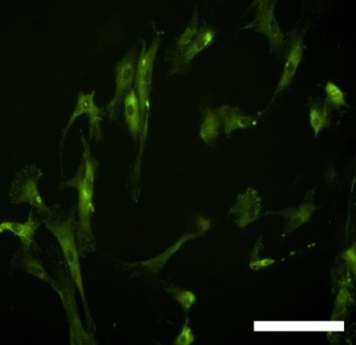


**Paxillin**


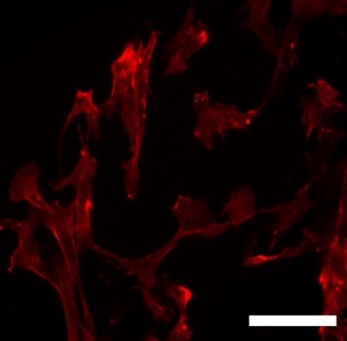


**Phalloidin**

**Supplementary Figure 1**. Immunocytochemistry staining for M0 cells at Day 2 in culture A) TCPS and B) FC40/DMEM interface. Green – anti-paxillin staining; Red – Phalloidin staining; Blue – DAPI. Scale bar represents 100 µm.

**
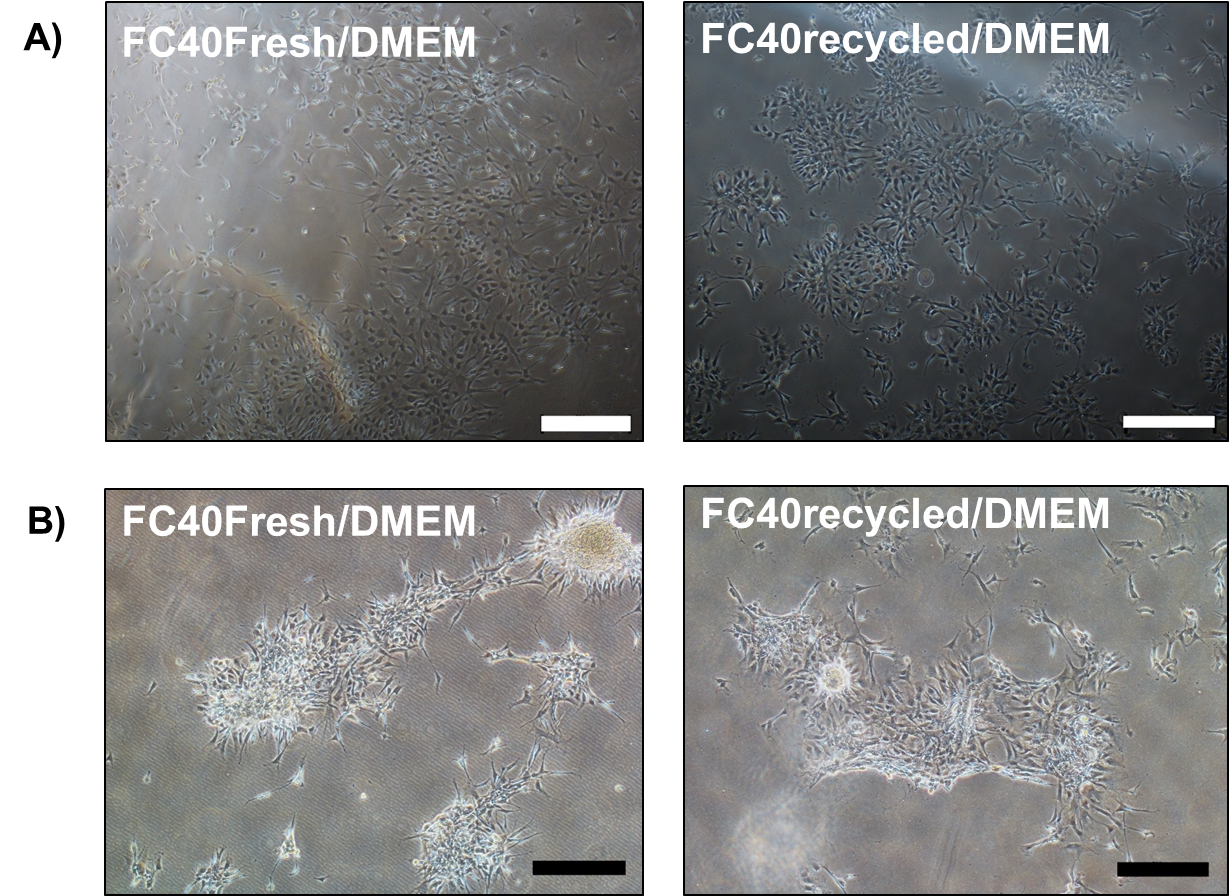
**

**Supplementary Figure 2**. Phase contrast images of A) M0 and B) M4 cell morphology at day 2 in culture on FC40fresh/DMEM or FC40recycled/DMEM interfaces. Scale bar represents 500 µm

B)

A)


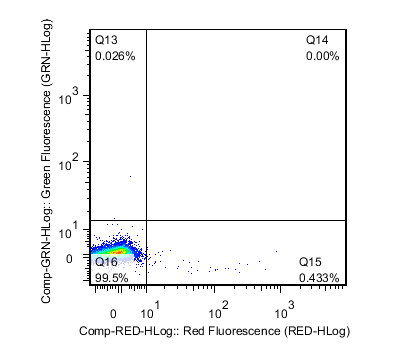

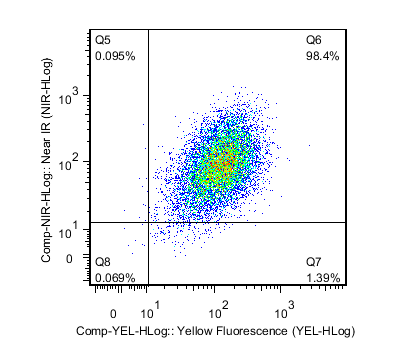

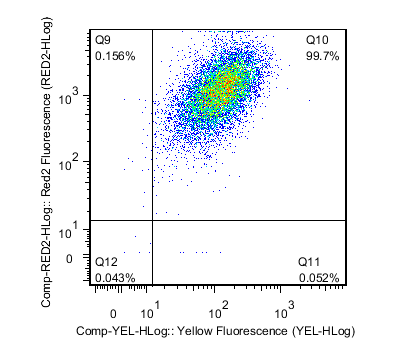

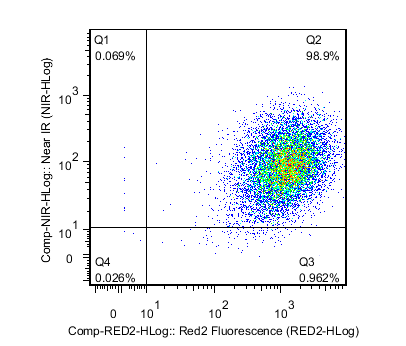


CD73/CD90

CD73/CD105

CD90/CD105

HLA-DR/CD34


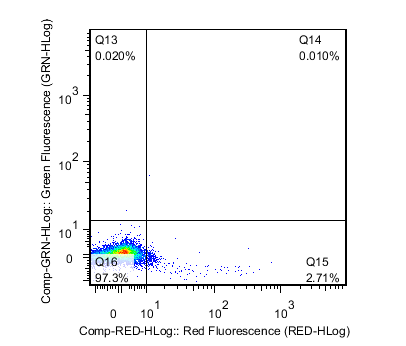

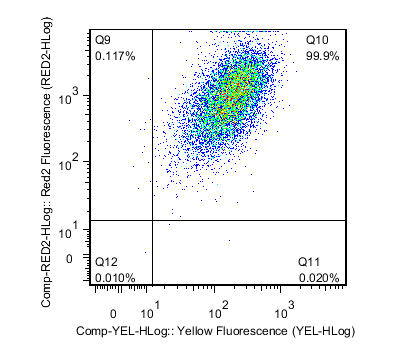

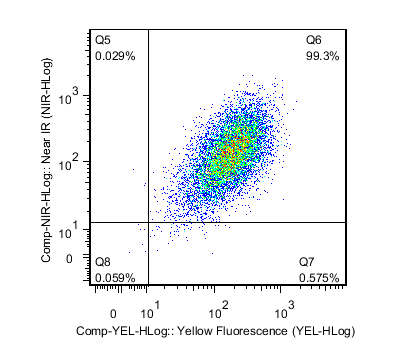

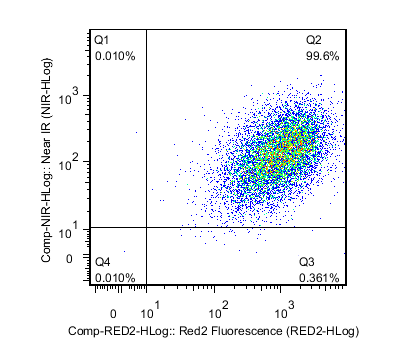


CD73/CD90

CD73/CD105

CD90/CD105

HLA-DR/CD34


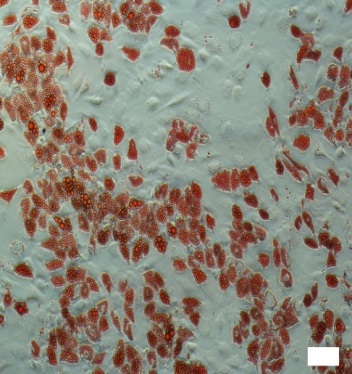


C)

**Pre-expansion**


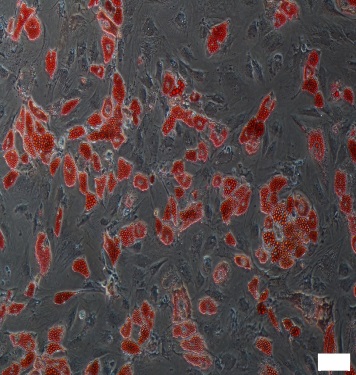


**Post-expansion**


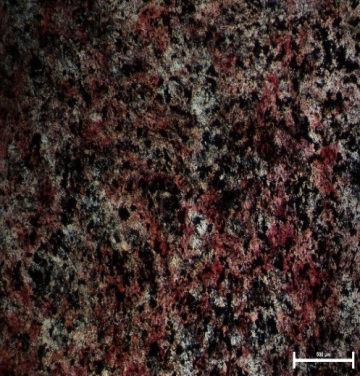


**Pre-expansion**

D)


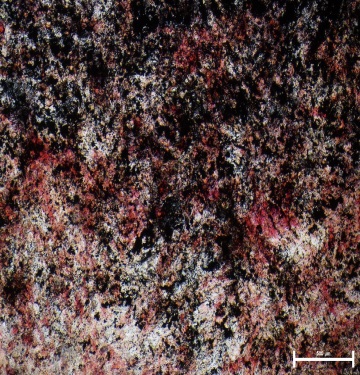


**Post-expansion**

**Supplementary Figure 3.** Cell surface markers expression of M0 cell line cultured on A) fresh and B) recycled FC40/DMEM interfaces, assessed by multi-parameter flow cytometry. M0 donor line differentiation towards C) adipogenic (Scale bar 100 µm) and D) osteogenic (Scale bar 500 µm) lineage.


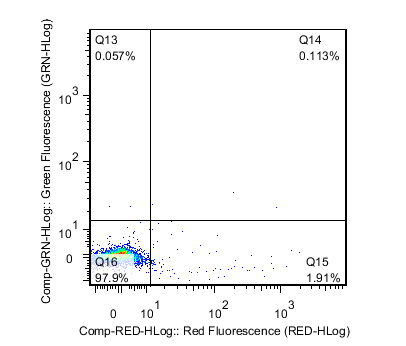

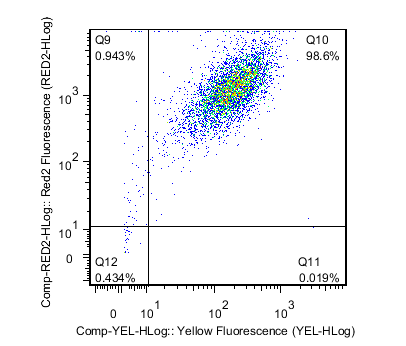

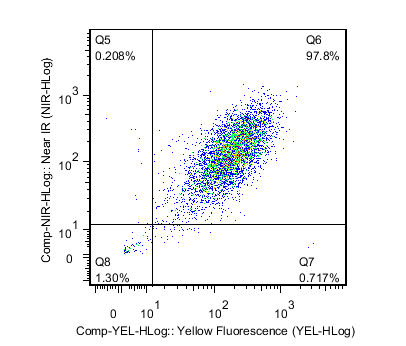

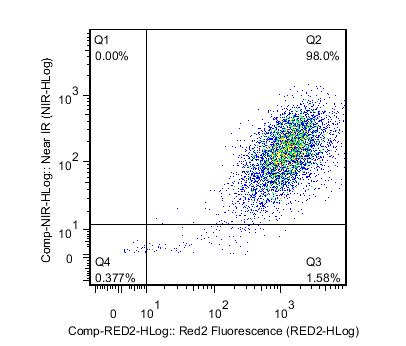


CD73/CD90

CD73/CD105

CD90/CD105

HLA-DR/CD34


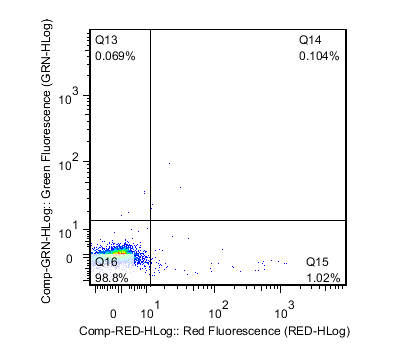

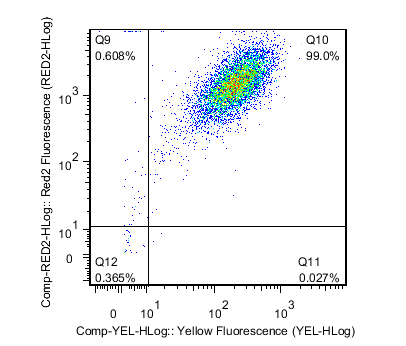

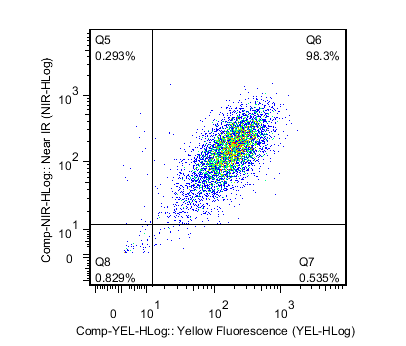

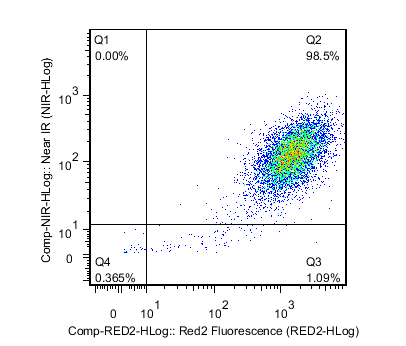


CD73/CD90

CD73/CD105

CD90/CD105

HLA-DR/CD34

A)

B)


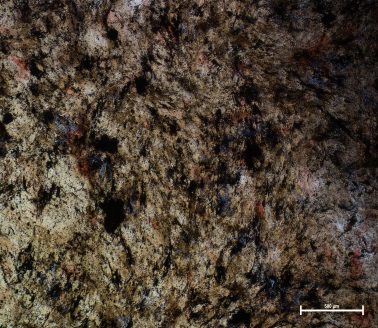

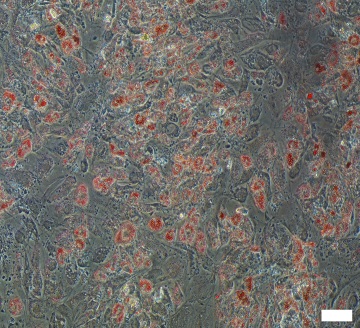

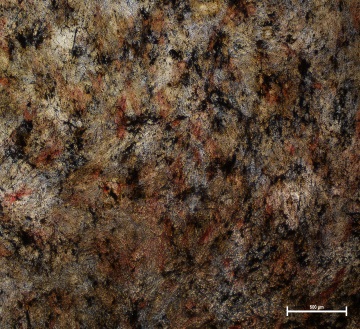

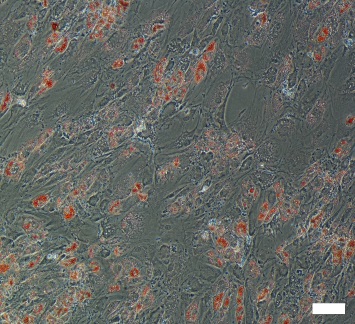


C)

**Pre-expansion**

**Post-expansion**

**Pre-expansion**

D)

**Post-expansion**

**Supplementary Figure 4.** Cell surface markers expression of M4 cell line cultured on A) fresh and B) recycled FC40/DMEM interfaces, assessed by multi-parameter flow cytometry. M4 donor line differentiation towards C) adipogenic (Scale bar 100 µm) and D) osteogenic (Scale bar 500 µm) lineage.
